# Biomass-Based Shape-Stabilized Composite Phase-Change Materials with High Solar–Thermal Conversion Efficiency for Thermal Energy Storage

**DOI:** 10.3390/polym15183747

**Published:** 2023-09-13

**Authors:** Ning Gao, Jiaoli Du, Wenbo Yang, Youbing Li, Ning Chen

**Affiliations:** 1 College of Materials Science and Engineering, Chongqing University of Technology, Chongqing 400054, China; 2 State Key Laboratory of Polymer Materials Engineering, Polymer Research Institute, Sichuan University, Chengdu 610065, China

**Keywords:** pine cone porous biomass carbon, shape-stabilized phase-change materials, solar–thermal conversion, thermal energy storage, energy-storing rigid polyurethane foam composites

## Abstract

To alleviate the increasing energy crisis and achieve energy saving and consumption reduction in building materials, preparing shape-stabilized phase-change materials using bio-porous carbon materials from renewable organic waste to building envelope materials is an effective strategy. In this work, pine cone porous biomass carbon (PCC) was prepared via a chemical activation method using renewable biomaterial pine cone as a precursor and potassium hydroxide (KOH) as an activator. Polyethylene glycol (PEG) and octadecane (OD) were loaded into PCC using the vacuum impregnation method to prepare polyethylene glycol/pine cone porous biomass carbon (PEG/PCC) and octadecane/pine cone porous biomass carbon (OD/PCC) shape-stabilized phase-change materials. PCCs with a high specific surface area and pore volume were obtained by adjusting the calcination temperature and amount of KOH, which was shown as a caterpillar-like and block morphology. The shape-stabilized PEG/PCC and OD/PCC composites showed high phase-change enthalpies of 144.3 J/g and 162.3 J/g, and the solar–thermal energy conversion efficiencies of the PEG/PCC and OD/PCC reached 79.9% and 84.8%, respectively. The effects of the contents of PEG/PCC and OD/PCC on the temperature-controlling capability of rigid polyurethane foam composites were further investigated. The results showed that the temperature-regulating and temperature-controlling capabilities of the energy-storing rigid polyurethane foam composites were gradually enhanced with an increase in the phase-change material content, and there was a significant thermostatic plateau in energy absorption at 25 °C and energy release at 10 °C, which decreased the energy consumption.

## 1. Introduction

With the rapid development of the global economy and rapid growth of the population, traditional energy resources have gradually decreased and the deterioration of the ecological environment caused by energy consumption has become increasingly apparent. Under this circumstance, there is an urgent need for the exploitation of new renewable energy to solve the energy crisis and eco-environment issues [1]. Phase-change materials (PCMs), as environmentally friendly energy-saving materials, can store and utilize thermal energy in the process of absorbing or releasing heat [2,3,4], further achieving the goal of energy conservation and emission reduction. Therefore, PCM has been widely applied in regulating textiles [5,6], intelligent buildings [7,8], solar energy storage [9,10], and other application fields [11,12]. At present, in spite of the fact that organic phase-change materials (polyethylene glycol, octadecane, n-heptadecane, and n-hexadecane) have been widely applied due to their sizeable latent heat value and small volume changes [13,14], the fluidity and easy leakage of the liquid phase existing in the phase-change process of the material limits its application, as well as its low heat conductivity [15,16]. Therefore, to solve the drawbacks mentioned above, porous-based supporting materials provide a thermally conductive framework while utilizing the capillary force generated by their high porosity and specific surface area to prevent PCM leakage, such as foam metal [17], graphite [18], carbon nanotubes [19], and porous carbon [20]. Among them, biomass-based porous carbon materials have attracted much attention in the field of porous carbon materials and have been widely applied in many fields, such as energy storage [21], supercapacitors [22], microwave absorption [23], and wastewater treatment [24], on account of their advantages of comprehensive sources, low cost, and renewability [15,25].

In order to reduce costs and maximize waste utilization, a large number of industrial and agricultural wastes such as corn cobs [26], hazelnuts shells [27], straw [28,29], walnut shells [30,31], coconut shells [32,33], durian shells [34], and industrial waste lignin [35] have been used to prepare biomass-based porous biomass carbons. The structure of biomass-based porous biomass carbon is a three-dimensional network composed of thin and defective graphene sheets. The pore structure includes a BET-specific surface area, average pore volume, and total pore size, which mainly depend on the structure of the biomass and the activation method used. The effective methods are physical and chemical activation methods. The physical activation methods form pores by reacting gas (steam, CO_2_, NH_3_, and mixture gases) with carbon atoms or other heteroatoms to generate CO, H_2_, and other gases, and the chemical activation methods treat biochar with alkali, acid, or metal salt to make pores, and the commonly used activators include KOH, NaOH, H_3_PO_4_, and ZnCl_2_ [36]. Luo et al. [37] obtained garlic peel porous biomass carbon (AGP) by activating garlic peel with KOH at a high temperature and developed a new shape stable phase-change material (SSPCM) for thermal energy storage with paraffin as the phase-change material. The results showed that AGP had many grooves and the specific surface area was 1309 m^2^/g. The phase-change enthalpies during melting and freezing were 52.5 J/g and 51.9 J/g, respectively, and SSPCM had a low latent heat loss rate of about 1.5% after 200 thermal cycles, indicating that the material had a good thermal cycle stability. Zhou et al. [38] prepared a hemp stalk three-dimensional porous biomass carbon (HSAC) with a high adsorption capacity using low-temperature hydrothermal carbonization combined with KOH high-temperature activation. A series of PEG/HSAC phase-change energy storage materials with different mass ratios were prepared via physical blending impregnation with polyethylene glycol (PEG) the phase-change material. The experimental results indicated that the maximum specific surface area of HSAC was 2310.1 m^2^/g. When the mass ratio of PEG to HSAC was 3:2, the latent heat of the composite was 51.5 J/g, the phase transition temperature was 58.5 °C, and there was no obvious leakage after 20 thermal cycles.

Pine cones are an agricultural waste composed of alpha-cellulose, hemicellulose, and lignin, which can be used as the supporting skeleton of a loaded phase-change material after carbonization due to their great pore structure. However, the material obtained by direct carbonization still maintains the original skeleton of pine cones after adsorbing a phase-change material, which limits its application ability in engineering. Therefore, it is an effective solution to obtain three-dimensional porous pine cone powder support skeleton material using chemical activation.

In this work, pine cone porous biomass carbon (PCC) was prepared with pine cones as a raw material and KOH as the activator. Two kinds of shaped composite phase-change materials, polyethylene glycol/pine cone porous biomass carbon (PEG/PCC) and octadecane/pine cone porous biomass carbon (OD/PCC), were synthesized using the vacuum impregnation method. The pore size and surface properties of the PCC were adjusted by the synergistic effect between the calcination temperature and KOH. The prepared PCC was used as a supporting material to prevent PCM leakage and improve the thermal conductivity and photothermal conversion ability through its capillary force, and the heat storage properties of PEG/PCC and OD/PCC were studied in detail. In addition, the effects of the PEG/PCC and OD/PCC additions on the temperature-regulating and temperature-controlling capabilities of energy-storing rigid polyurethane foams were investigated.

## 2. Experimental Methods

### 2.1. Materials

Pine cones were collected from a pine plantation area of Luoyang, Henan Province (China). Potassium hydroxide (KOH, A.R.), hydrochloric acid (HCl, A.R.), and ethanol (A.R.) were obtained from Chengdu Kelong Chemical Reagent Co., Ltd. (Chengdu, China). Polyethylene glycol (PEG, Mn = 4000) was provided by Bayer Co., Ltd. (Shanghai, China). Octadecane (OD) was purchased from Shanghai Aladdin Bio Chem Technology Co., Ltd. (Shanghai, China).

### 2.2. Preparation of Pine Cone Porous Biomass Carbon

The PCC was prepared using the chemical activation method. First, the pine cones were washed thoroughly by deionized water and anhydrous ethanol, then dried under vacuum at 60 °C for 48 h. The dried pine cones were powdered using an electric grinder machine and sieved through a 0.5 mm sieve. KOH was used as an activator, and 1.5 g of pine cone powder was ground with KOH at mass ratios of 1:1, 1:2, 1:3, and 1:4, then heated to 700 °C, 800 °C, and 900 °C at 10 °C/min in a tube furnace N_2_ atmosphere, and pyrolyzed at these temperatures for 1.5 h. The carbonized material was soaked in excess 1 mol/L HCl for 10 h to remove excess KOH, and then washed with ethanol and deionized water and placed in a 60 °C oven for 12 h. All the PCC-X-Y samples are shown in Table 1; the X corresponds to the different mass ratios of KOH and Y corresponds to the different calcination temperatures.

### 2.3. Preparation of Composite Form-Stable PCMs

The form-stable PEG/PCC and OD/PCC were synthesized using the vacuum impregnation method. The PCC was dried in a vacuum oven at 80 °C for 4 h, and then polyethylene glycol and octadecane were melted at 80 °C. The dried PCC was added to the two solutions and then placed in an 80 °C vacuum oven at 0.08 MPa for 12 h. Finally, the material was placed on the filter paper and repeatedly heated at 80 °C for 20 min until the filter paper showed no leakage. The two composite form-stable phase-change materials were taken out at room temperature.

### 2.4. Preparation of Rigid Polyurethane Foam for Energy-Saving Building

**Figure 1:**
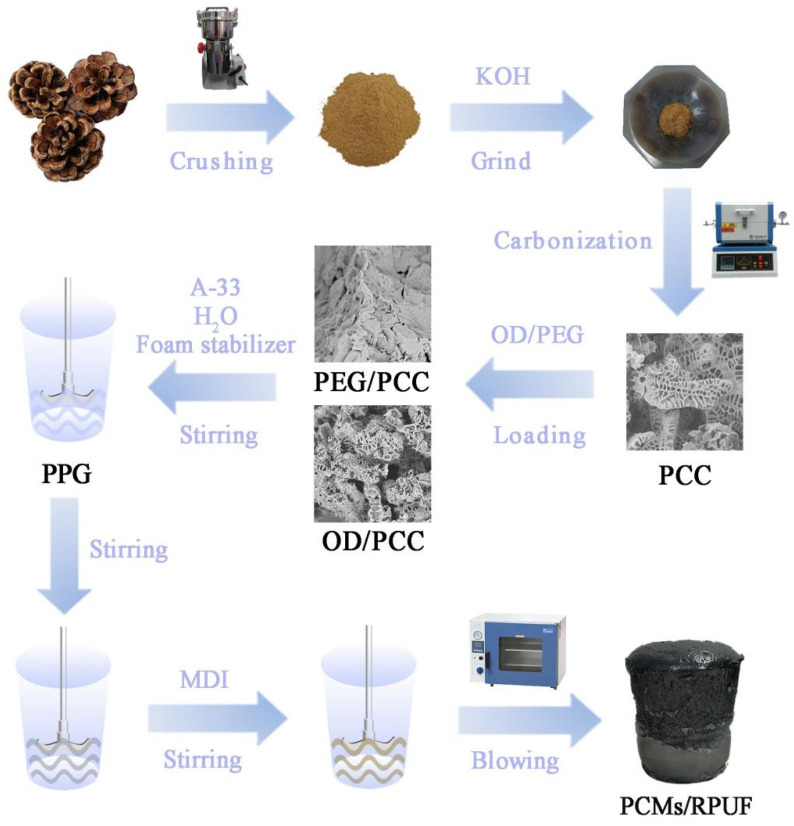
The schematic diagram of PCMs/RPUF composite preparation process.

Using water as a blowing agent, triethylenediamine as a catalyst, and OD/PCC and PEG/PCC as functional materials, a fully water-blown rigid polyurethane foam for energy storage was prepared using a one-step method. An appropriate amount of dry PPG was poured into a plastic cup, and then reasonable amounts of foam stabilizer, blowing agent, and catalyst were added and stirred with a stirrer at a speed of 1200 r/min for 2 min to obtain a well-mixed white material. OD/PCC and PEG/PCC materials with different mass fractions in a mass ratio of 1:1 were added to the white material and stirred with a stirrer at a speed of 1200 r/min for 5 min to make OD/PCC and PEG/PCC disperse in the white material. The specific sample composition formula is shown in Table 2. Furthermore, a fair amount of MDI was added to the white material, stirred with a stirrer at a speed of 1500 r/min, which was stopped after the plastic cup heated up, and the material was put in a 30 °C oven to freely foam and mature for 24 h. The synthetic route of PCMs/RPUF is briefly sketched in Figure 1.

### 2.5. Characterizations

The morphologies and microstructures of the PCCs and shape-stabilized PCMs were observed using field emission scanning electron microscopy (SEM, JSM-5900LV, Tokyo, Japan). The condensed-phase structures of the PCCs and shape-stabilized PCMs were investigated using X-ray diffraction (XRD, Ultima IV, Japan, Cu Ka radiation at 40 kV and 50 mA). The chemical structures of the PCCs and shape-stabilized PCMs were analyzed using Fourier transform infrared spectroscopy (FT-IR, Nicolet 50, Madison, WI, USA) and the infrared Wavenumber from 4000 to 400 cm^−1^. The porous structures of the PCCs were evaluated through Brunauer–Emmett–Teller (BET, Belsorp-Max, Shanghai, China). The graphitization degrees of the PCCs were studied using Raman spectra (inVia Reflex, London, UK). The thermal decomposition behavior of the PCCs and shape-stabilized PCMs was studied using a thermogravimetric analysis (TGA, TGA 2, Zurich, Sweden) from 35 to 800 °C at a heating rate of 10 °C/min under a N_2_ atmosphere. The storage capacities of all the shape-stabilized PCMs and PCMs/RPUF composites were characterized by differential scanning calorimetry (DSC, TA Q250, New Castle, DE, USA) at a heating/cooling rate of 10 °C/min under a N_2_ atmosphere. The solar thermal energy conversion capability of the two shape-stabilized PCMs was evaluated via an infrared thermal imager (FTLR T420, Shanghai, China). The thermal conductivity of the two shape-stabilized PCMs and PCMs/RPUF composites was studied using a transit hot disk thermal constant analyzer (Hot disk 2500-OT, Uppsala, Sweden). The shape stability of the shape-stabilized PCMs was examined by photographing the leakage of the samples before and after 30 min at 80 °C. The mechanical properties of the PCMs/RPUF composites were tested with an electronic universal tensile machine (Instron 68TM-10, Norwood, MA, USA) at a test speed of 5 mm/min on samples with a size of 50 mm × 50 mm × 50 mm, and the deformation reached 10%. The thermoregulation and temperature control ability of the PCMs/RPUF composites were tested using a high-precision thermocouple multi-channel temperature tester [10], which was set to heat from 0 °C to 90 °C at a rate of 0.65 °C/min and cool from 90 °C to 0 °C at a rate of 0.3 °C/min.

## 3. Results and Discussion

### 3.1. Structure of PCCs

**Figure 2:**
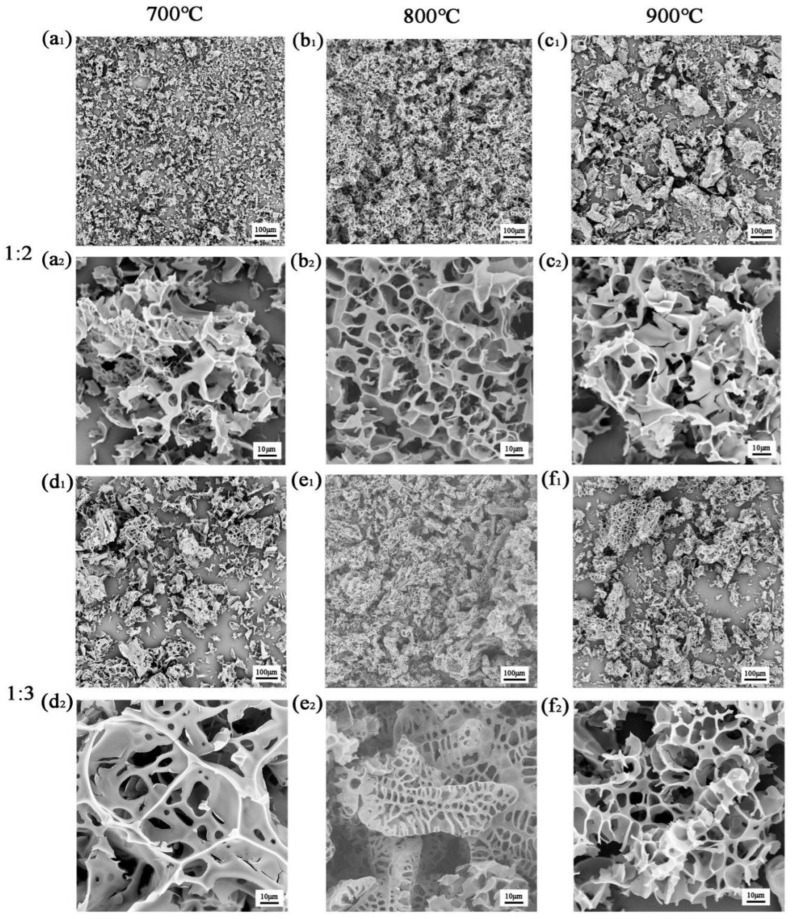
SEM images of (**a_1_**,**a_2_**) PCC-2-700, (**b_1_**,**b_2_**) PCC-2-800, (**c_1_**,**c_2_**) PCC-2-900, and (**d_1_**,**d_2_**) PCC-3-700, (**e_1_**,**e_2_**) PCC-3-800, and (**f_1_**,**f_2_**) PCC-3-900.

Three-dimensional interpenetrated PCC materials were prepared via high-temperature calcination using pine cones as the carbon-rich raw material and KOH as the activator. The pore structures of the fabricated PCCs were adjusted by controlling the KOH content and calcination temperature. Figure S1 and Figure 2(b1,b2,e1,e2) show the effects of the different KOH contents on the pore structures of the PCCs at a calcination temperature of 800 °C. As shown in Figure S1a, the pine cone itself had a good pore structure with a pore size between 20 and 50 μm, which is ideal for a pore-forming biological raw material, and the morphology of the pine cone was mainly in fragments when it was broken. When the mass ratio of pine cones to KOH was 1:1, as shown in Figure S1b, a new pore structure started to form through the activation of KOH. When the mass ratio of pine cones to KOH was 1:2, as shown in Figure 2(b1,b2), it could be clearly seen that the activation of KOH led to the formation of a relatively homogeneous three-dimensional pore structure on the surface of the sample with a large pore size of about 10–20 μm. When the mass ratio of pine cones to KOH was 1:3, as shown in Figure 2(e1,e2), a three-dimensional void structure, similar to the morphology of caterpillars, appeared due to the further activation of KOH for the rod-shaped fibers of the pine cones, along with a blocky three-dimensional void structure which was more similar to the pore structure when the pine cone was not activated and when the mass ratio of pine cone to KOH was 1:2, showing different pore structures with sizes of mainly 5–15 μm. When the mass ratio of pine cones to KOH was 1:4, as shown in Figure S1c, the carbon skeleton was washed out into fragments of different sizes and shapes, because the reaction was more intense with a higher KOH content. Therefore, when the mass ratios of pine cones to KOH were 1:2 and 1:3, a great three-dimensional interpenetrating network structure could be formed. Combined with Figure 2 and Figure S1, the pores formed at a 1:3 pine cone to KOH mass ratio were more uniform and perfect than the sample with a 1:2 mass ratio. At an 800 °C calcination temperature, the samples with a 1:3 mass ratio of pine cones to KOH had a smaller pore size and more visible open pores inside than the 1:2 samples, due to the fact that the higher concentration of KOH during activation led to a strong chemical etching process on the carbon atoms and created a more porous surface for the sample.

Figure 2 shows the effect of the different calcination temperatures on the pore structures of the PCC materials at pine cones to KOH mass ratios of 1:2 and 1:3. When the calcination temperature was 700 °C, there were some micro-pores of irregular sizes and shapes in the stacked skeleton structure, but the yield was relatively low (Figure 2(a1,a2)). When the calcination temperature was increased to 800 °C, a three-dimensional network pore structure with a high yield and relatively uniform shape and size was formed (Figure 2(b1,b2)). When the calcination temperature was further increased to 900 °C (Figure 2(c1,c2)), the KOH activation strength was stronger and a large number of three-dimensional network structures were washed out, although the size and shape of the pore structure were relatively uniform, a larger pore size structure was formed, the yield was reduced relative to that at 800 °C, and the three-dimensional network structure was cracked and easily broken.

**Figure 3:**
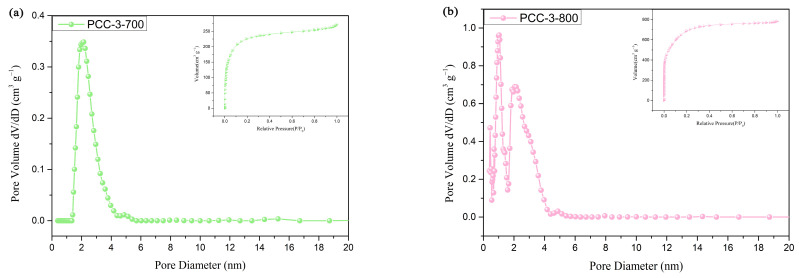

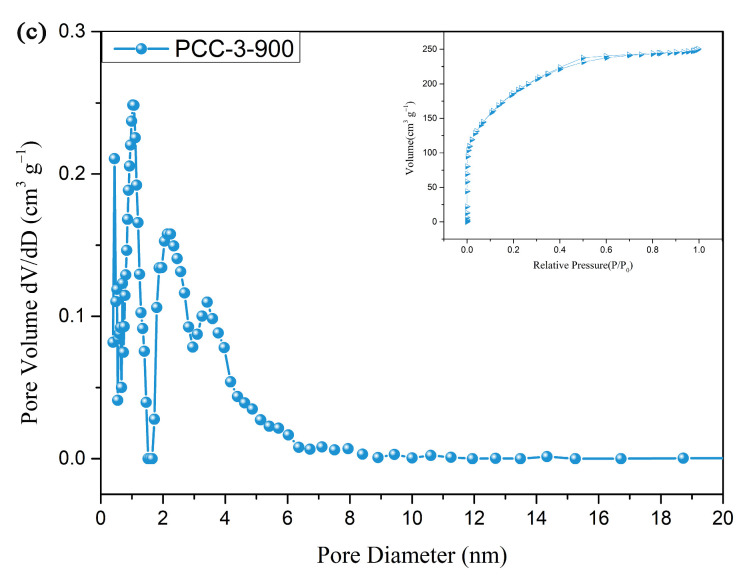
DFT desorption pore size distribution and nitrogen adsorption/desorption isotherms (inset) of (**a**) PCC-3-700, (**b**) PCC-3-800, and (**c**) PCC-3-900.

To further evaluate the pore structure and adsorption capacity of the PCC, the specific surface area and pore size of the PCC were conducted using N_2_ adsorption–desorption isotherms, as shown in Figure 3 and Figure S2. As shown in Figure S2a, PCC-2-800 was a type I isotherm according to the IUPAC classification, which indicated that the pore structure was microporous when the pine cones to KOH mass ratio was 1:2. Combined with Figure S2b and Table 3, it further indicated that no mesopores existed in this material and the average pore size of the micropores was 1.58 nm. According to the IUPAC classification, the isotherms of both PCC-3-700 (Figure 3a) and PCC-3-800 (Figure 3b) were a combination of type I and type II. The type I adsorption isotherm indicated a microporous material, and the type II adsorption isotherm represented the presence of mesopores, thus manifesting the coexistence of micro and mesopores in both materials [39,40]. When the type I isotherm curve did not reach equilibrium at a lower relative pressure (around 0.1), the material may have had a large number of narrower mesopores with a pore width less than 2.5 nm, in addition to the presence of a large number of micropores, combined with Figure 3a,b and Table 3, which show that the pore structures of PCC-3-700 and PCC-3-800 were micropore–mesopore. Figure 3c shows that PCC-3-900 had an H4 lagging backline with type IV isotherms at higher relative pressures (0.4 < P/P0 < 0.99), which indicated the presence of mesopores. As can be seen from Table 3, with an increase in the calcination temperature, the specific surface area of the samples first increased from 862.04 m^2^/g to 1758.6 m^2^/g and then decreased to 679.73 m^2^/g, the pore volume first increased from 0.42 cm^3^/g to 1.21 cm^3^/g and then decreased to 0.38 cm^3^/g, and the average pore size changed from 1.93 nm to 2.74 nm and then decreased to 2.26 nm. As the same time, the gas adsorption was also smaller for PPC-3-700 and PCC-3-900 compared to PCC-3-800. This was because, as the calcination temperature increased from 700 °C to 800 °C, the activation of KOH increased, and the PCC formed a complete pore structure. A further increase in the temperature to 900 °C caused a more severe erosion of the PCC, and the rupture of the micro-pore walls and the formation of mesopores were faster than the creation of new pores, which led to the collapse of the pore structure. The results were compared with the various other carbon materials in Table 3 and Table S1, and the BET surface area and pore volume of PCC-3-800 were excellent, which was beneficial for the loading of phase-change materials.

**Figure 4:**
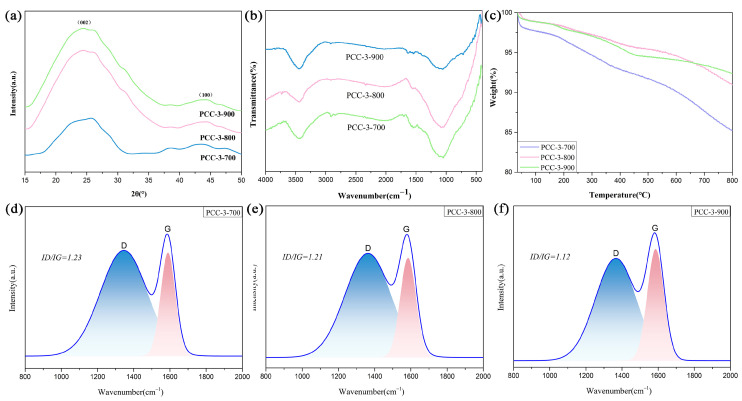
(**a**) X-ray diffraction analysis of PCC-3-Y, (**b**) FTIR spectra of PCC-3-Y, (**c**) TG curves of PCC-3-Y, and (**d**–**f**) Raman spectra of PCC-3-Y (Y = 700, 800, 900).

Combining Figure 2 and Table 3, it is clear that the specific surface area of the pore structure of the PCC increased and then decreased with an increase in the carbonization temperature, with a partial shift from micropores to mesopores, which was attributed to the rearrangement between the lamellar structures of the PCC material due to the increase in the calcination temperature under the effect of KOH activation. The stacking state and degree of graphitization of the activated carbon were examined using XRD. According to Bragg’s equation (nλ = 2dsinθ), the 2θ (about 25°) values of all the samples were lower than the standard graphite value (26.6°), indicating the widening/broadening of the layer spacing [15,44]. As shown in Figure 4a, the XRD patterns of PCC-3-800 and PCC-3-900 had two diffraction peaks at 24.5° and 44°, corresponding to the disordered graphite planes (002) and graphite crystal plane (100) diffraction plane. The former plane (002) demonstrated that the carbon in the sample was in an amorphous state due to the rearrangement stacking reaction, which may have contributed to the formation of microporous channels, and the latter plane (100) indicated the degree of graphitization possessed by the material. The two diffraction peaks of PCC-3-800 and PCC-3-900 were more robust compared to the two diffraction peaks of PCC-3-700; therefore, increasing the calcination temperature could improve the crystallinity and graphitization of the biomass carbon. A Raman analysis was performed to elucidate the graphitization of the porous carbon further. The Raman spectra in Figure 4d–f show broad peaks at 1362 cm^−1^ and 1576 cm^−1^, corresponding to the D and G bands of the carbon, respectively. The D band represents disordered sp^2^ hybridized carbon atoms, and the G band is associated with the phonon mode of graphite and the formation of graphitic carbon [45]. The relative intensity ratio (ID/IG) of the integrated area of the PCC material decreased from 1.23 to 1.12 as the calcination temperature increased, which implied an increase in the graphitic structure [15,46,47]. At the same time, the presence of an appropriate graphite content in the samples improved the strength and thermal conductivity of the PCC, thus improving its suitability as a framework material for phase-change materials as thermal energy storage materials.

The FTIR spectrum of the PCC in Figure 4b revealed that the absorption peak at the 3430 cm^−1^ broad peak corresponded to the -OH stretching vibrations in alcohols, phenols, and carboxyl functional groups. The absorption peak at 2920 cm^−1^ was attributed to the asymmetric and symmetric stretching vibrations of the -CH_2_ groups in aliphatic hydrocarbons or cycloalkanes. The vibration absorption peak at 1570 cm^−1^ referred to the conjugated C=C double bond on the benzene ring, and the band at 1060 cm^−1^ testified to the stretching vibration of C-O in aliphatic ethers. The thermal stability of the PCC materials is shown in Figure 4c and Table S2. It could be observed that the first thermal weight loss stage occurred mainly before 200 °C, which was mainly manifested by the evaporation of the water physically adsorbed by the PCC. The second and third thermal decomposition stages were distributed at 200–470 °C and 470–800 °C, respectively, and the weight loss originated from the thermal decomposition of the residual organic components in the PCC, which further removed the oxygen-containing groups and generated free radical fragments eventually [15,48,49]. The generated free radical fragments were finally generated into stable small molecules such as CO, CO_2_, and H_2_O. Furthermore, the amount of stabilized carbon increased as the calcination temperature increased from 700 °C to 900 °C, which led to an increasing thermal stability of the PCC material with weight losses of 14.8%, 9.1%, and 7.6%, respectively.

### 3.2. Formation Mechanism

**Figure 5:**
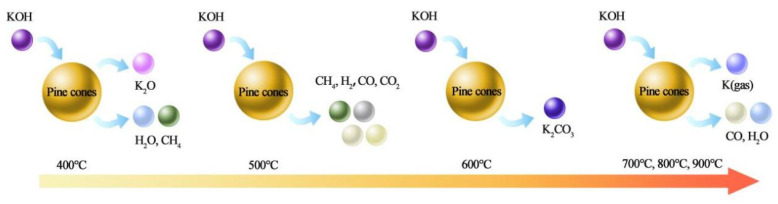
Mechanism diagram for the formation process of PCC materials.

The carbonation activation mechanism of the PCC with temperature change is shown in Figure 5. According to Figure 4c, when the calcination temperature was no more than 200 °C, the water inside the pine cone material evaporated into water vapor, including physically adsorbed water, free water, and combined water. When the calcination temperature was 200–500 °C, with an increase in the calcination temperature, KOH started to decompose into K_2_O and H_2_O, and hemicellulose, cellulose, and lignin produced a large amount of gas CH_4_ [48], CO, CO_2_, and H_2_O through pyrolytic treatment, then CO and CO_2_ with a H_2_O secondary reaction produced a large amount of H_2_, respectively. The carbon skeleton, after the initial heat treatment carbonization, was formed in the process of gas release and a collision reaction. Part of the K_2_O and CO_2_ reacted to obtain K_2_CO_3_ when the temperature was increased to 600 °C. When the calcination temperature was 700–900 °C, the K_2_O and K_2_CO_3_ evolved from KOH further etched the carbon skeleton to release a lot of gases, including K (gas), CO, and H_2_O, etc. These gases further impacted the carbon skeleton in the release process and obtained a three-dimensional interpenetrating network skeleton structure with better pores. Compared to the pore structure at the calcination temperature of 800 °C, when the calcination temperature was 700 °C, the reaction of K_2_O and K_2_CO_3_ evolved from the KOH etching carbon skeleton was incomplete, the production of gas was relatively less, and the pressure for its punching through part of the pore wall was insufficient, so the pore structure of the formed three-dimensional interpenetrated network skeleton structure was not uniform and the yield was relatively low. When the calcination temperature was 900 °C, the K_2_O and K_2_CO_3_ reacted perfectly with the carbon skeleton and produced relatively more gas and a higher pressure for its punching part of the pore wall; thus, the pore wall was easy to break and the sufficient reaction made part of the carbon skeleton collapse, which led to the formation of a three-dimensional interpenetrated pore structure, which was uniform but with a lower yield. The equations illustrating the above processes are presented below.

(1) $$2KOH\to K_{2}O+H_{2}O$$

(2) $$C+H_{2}O\to H_{2}+CO$$

(3) $$CO+H_{2}O\to H_{2}+CO_{2}$$

(4) $$K_{2}O+CO_{2}\to K_{2}CO_{3}$$

(5) $$K_{2}O+H_{2}\to 2K+H_{2}O$$

(6) $$K_{2}O+C\to 2K+CO$$

(7) $$K_{2}CO_{3}+2C\to 2K+3CO$$

### 3.3. Structures of Composite PCMs

**Figure 6:**
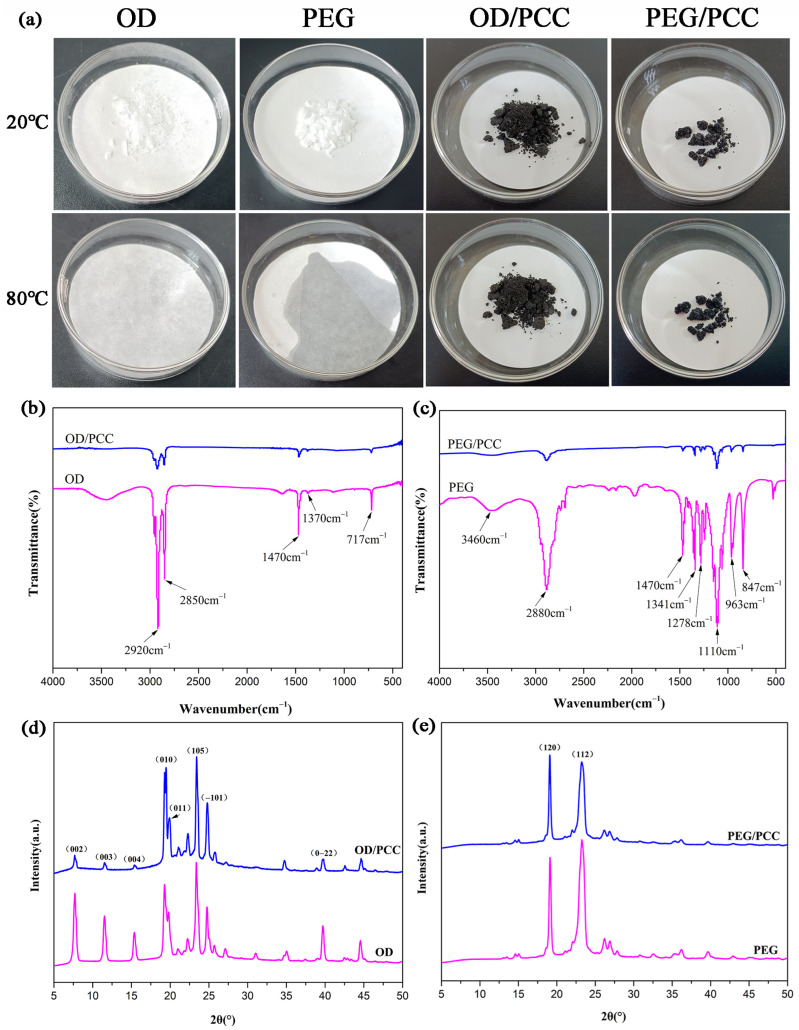
(**a**) The form-stabilized ability of pure octadecane and related composite PCMs at room temperature (20 °C) and above the melting temperature (80 °C), (**b**,**c**) FTIR spectra, and (**d**,**e**) X-ray diffraction curves of OD, OD/PCC, PEG, and PEG/PCC.

Combined with the above analysis, it was apparent that PCC-3-800 was the ideal carbon material for loading phase-change materials. The two shape-stabilized OD/PCC and PEG/PCC were obtained via the direct impregnation of OD and PEG into PCC-3-800 in a vacuum oven. As shown in Figure S3, there were almost no open pores in PEG/PCC, indicating that the pores of PCC-3-800 were fully filled by PEG. Furthermore, the relative PEG/PCC was overtly observed in OD/PCC, and OD did not completely fill the PCC pores due to the lower molecular weight of octadecane compared to that of polyethylene glycol. When the temperature rose to 80 °C, the mobility of the octadecane molecules was better than that of polyethylene glycol, with the larger pores of PCC-3-800 mainly playing the role of transport pipeline, and the loading of OD mainly being due to the capillary force of the microporous–mesoporous structure. In order to test the shape stabilization ability of OD/PCC and PEG/PCC, a temperature of 80 °C, which ensured that both OD and PEG could melt, was used to heat for 30 min and was verified by observing the dry and wet state of the filter paper, as shown in Figure 6a, when, at room temperature of 20 °C, the OD, PEG, OD/PCC, and PEG/PCC were all solid state. When heated to 80 °C and maintained for 30 min, the OD and PEG were transformed into liquid states, the OD/PCC and PEG/PCC were still solid states, and the filter paper did not appear wet, which indicated that the OD/PCC and PEG/PCC were two kinds of shape-stable composite phase-change materials.

The interaction mechanism of the PCC-3-800-loaded OD and PEG was verified by FT-IR. Figure 6b shows the infrared spectra of OD and OD/PCC. The peaks induced by 2920 cm^−1^ and 2850 cm^−1^ in OD represented the saturated C-H stretching vibration of aliphatic. The infrared peaks at 1470 cm^−1^ and 1370 cm^−1^ referred to the C-H bending vibration of methylene and the symmetrical bending vibration of methyl, respectively. The infrared peak at 717 cm^−1^ corresponded to the in-plane rocking peak of methylene [50,51]. Figure 6c shows the infrared spectra of PEG and PEG/PCC. The broad absorption peak at 3460 cm^−1^ in PEG was attributed to the stretching vibration of O-H, while the absorption peak at 1110 cm^−1^ was attributed to the C-O-C symmetric stretching vibration. In addition, the characteristic absorption peaks at 2880 cm^−1^, 1470 cm^−1^, 1341 cm^−1^, 1278 cm^−1^, 963 cm^−1^, and 847 cm^−1^ corresponded to the C-H vibration. The absence of new characteristic peaks in OD/PCC and PEG/PCC indicated that the interaction between OD, PEG, and PCC-3-800 was physical adsorption. The weakening of the corresponding peaks in OD/PCC and PEG/PCC implied that the octadecane molecules and polyethylene glycol molecules were adsorbed by the PCC-3-800 network pore structure under the action of capillary and surface tensions, thus preventing them from leaking from PCC-3-800 during the solid–liquid phase transition [52,53].

The crystallization behaviors of OD/PCC and PEG/PCC were analyzed using XRD, as shown in Figure 6d,e. The diffraction peaks of OD/PCC at 2θ = 7.78°, 11.54°, 15.38°, and 39.68° corresponded to (002), (003), (004), and (0-22), respectively, which showed a weakening trend compared to pure octadecane, while with the diffraction peaks of OD/PCC at 2θ = 19.48°, 19.94°, 23.42°, and 24.82°, four high-intensity diffraction peaks could be clearly observed at (010), (011), (105), and (-101), showing an enhanced trend compared to pure octadecane. Apparently, some of the α crystals of octadecane were transferred to β crystals after the OD impregnation into PCC-3-800, which indicated that the PCC-3-800 reticular pore structure enhanced the integrity of the crystallization of the β crystals of octadecane and caused a heterogeneous nucleation effect [15,54]. The high-intensity diffraction peaks of PEG/PCC at 2θ = 19.08° and 23.18° represented the characteristic crystal planes (120) and (112) [4,55,56,57], which reversed the intensity of the peak compared to that of the pure polyethylene glycol, suggesting that the PCC-3-800 reticular pore structure enhanced the crystalline integrity of the polyethylene glycol for crystal plane (120) and induced a heterogeneous nucleation effect. All the other weak peaks of PEG/PCC showed a weakening trend, which implied that the presence of PCC-3-800 had a limiting effect on the crystallization of the polyethylene glycol. Meanwhile, no new diffraction peaks were generated in the OD/PCC and PEG/PCC, which illustrated that the crystal structures of OD in OD/PCC and PEG in PEG/PCC were not changed, indicating that OD and PEG were adsorbed into the porous structure of PCC-3-800 by physical interaction, which was consistent with the FT-IR spectroscopy results.

### 3.4. Thermal Properties and Stability of Composite PCMs

**Figure 7:**
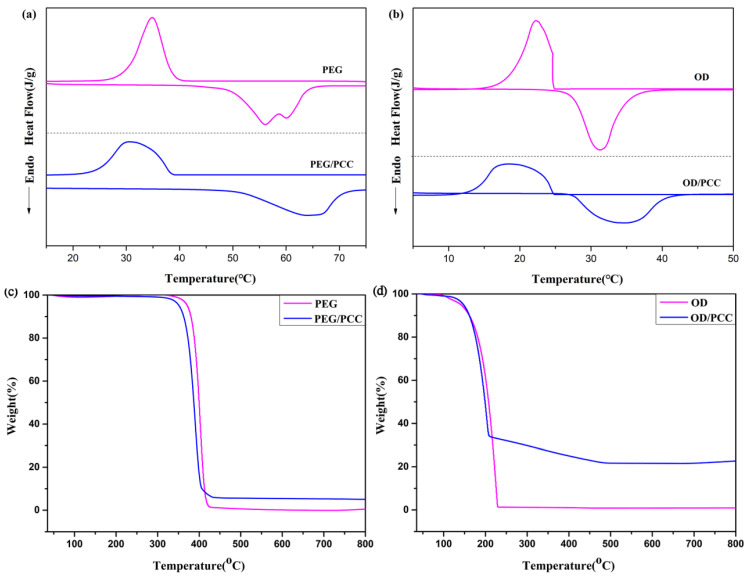
(**a**) DSC curves of PEG and PEG/PCC; (**b**) DSC curves of OD and OD/PCC; (**c**) TG curves of PEG and PEG/PCC; and (**d**) TG curves of OD and OD/PCC.

**Figure 8:**
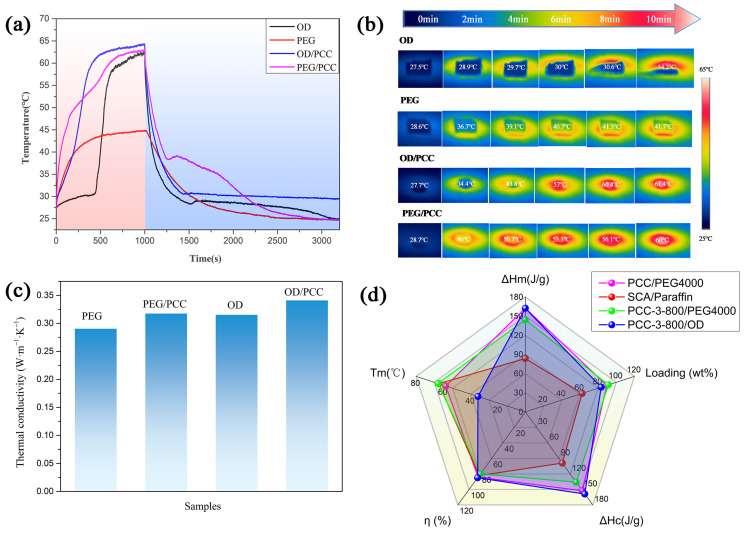
(**a**) Thermal transport evolution of OD, PEG, OD/PCC, and PEG/PCC during the heating process; (**b**) solar–thermal energy conversion and storage curves of OD, PEG, OD/PCC, and PEG/PCC under xenon lamp; (**c**) thermal conductivities of PEG, OD, PEG/PCC, and OD/PCC; (**d**) and Radar plot of the samples compared to other composite PCMs [64,65].

The thermal behaviors of OD/PCC and PEG/PCC were studied using DSC, and the energy storage capacity was reflected by the phase-change temperature and enthalpy. The results are shown in Figure 7a,b and Table 4. The melting enthalpy and crystallization enthalpy of OD were 194.5 J/g and 193.3 J/g, respectively, and the melting temperature and crystallization temperature were 27.5 °C and 24.6 °C, respectively. The melting enthalpy and crystallization enthalpy of PEG were 157.8 J/g and 151.9 J/g, respectively, and the melting temperature and crystallization temperature were 56.1 °C and 34.9 °C, respectively. Compared to pure OD and PEG, the enthalpies of OD/PCC and PEG/PCC decreased, and the melting enthalpies were 162.3 J/g and 144.3 J/g, respectively, which was attributed to the addition of PCC-3-800 reducing the mass ratio of OD and PEG and the physical adsorption of PCC-3-800 limiting the movement of OD and PEG during the phase transition. In addition, OD/PCC and PEG/PCC exhibited higher melting temperatures and lower crystallization temperatures, resulting in a wider melting and crystallization temperature range due to the strong interaction between PCMs (OD and PEG) and PCC-3-800 [58,59]. Therefore, the interaction between PCMs and PCC-3-800 had a significant effect on the crystallization/melting behavior of the PCMs. The energy storage capacity of OD/PCC and PEG/PCC was further evaluated using Equation (8) to calculate the encapsulation ratio (R) [60,61,62].

(8) $$R=\frac{\Delta H_{m,PCMs/PCC}}{\Delta H_{m,PCMs}}\times 100\%$$

where $\Delta H_{m,PCMs}$ is the melting enthalpy of the pure OD and PEG and $\Delta H_{m,PCMs/PCC}$ is the melting enthalpy of the prepared shape-stabilized OD/PCC and PEG/PCC. The results in Table 4 were compared with the other composite phase-change materials in Table S3 and Figure 8d, and the loadings of OD and PEG in PCC-3-800 were as high as 83.4% and 91.4%, respectively, which implied that OD/PCC and PEG/PCC had an excellent heat storage capacity.

Thermal cycling tests were performed using DSC to estimate the cyclic stability of OD/PCC and PEG/PCC, and the results of 50 cycle tests, as shown in Figure S4, disclosed that the DSC curves of OD/PCC and PEG/PCC after 50 cycles of heating and cooling were basically consistent with those in the first cycle. The latent heat of OD/PCC and PEG/PCC after 50 thermal cycles lost only 0.23 J/g and 0.12 J/g, respectively, compared to the first cycle, which illustrated that both OD/PCC and PEG/PCC had an excellent thermal cycle stability and thermal reliability in terms of thermal energy storage and release.

Thermal stability is an essential factor in evaluating the practical use value of a material. The TG curves of PEG and PEG/PCC are shown in Figure 7c, and the TG curves of OD and OD/PCC are shown in Figure 7d. The pure polyethylene glycol and octadecane had only one thermogravimetric behavior, with initial thermal decomposition temperatures of 372 °C and 141 °C, respectively, when the mass loss was 5%, indicating that the polyethylene glycol samples had a great thermal stability. Both PEG/PCC and OD/PCC had two thermal weight loss behaviors, the first pyrolysis stage dominated by the pyrolysis of the PCMs and the second pyrolysis stage by the pyrolysis of the residual organic components in the PCC. When the mass loss was 5%, their initial thermal decomposition temperatures were 352 °C and 142 °C, respectively, indicating that PEG/PCC still had a good thermal stability, while the initial thermal decomposition temperature of OD/PCC was almost unchanged compared to that of the pure OD. According to Table S4, there were no residues of pure PEG and octadecane, and the residues of PEG/PCC and OD/PCC at 800 °C were 5.1% and 22.7%, respectively, which obviously showed that the PEG/PCC and OD/PCC materials had a higher thermal stability than the pure PEG and octadecane. Meanwhile, the thermal weight loss of the first phase of PEG/PCC and OD/PCC almost corresponded to their loading rates.

### 3.5. Thermal Conductivity and Solar–Thermal Energy Conversion

Thermal conductivity is an important thermal parameter for evaluating the rate of heat storage and release of composite phase-change materials during melting and freezing. The overall heat conduction velocity and heat transfer capacity of the composite material were reflected via a hot plate analysis and measurement. As shown in Figure 8c, the thermal conductivities of the pure PEG and OD were 0.2907 W/(m·K) and 0.3156 W/(m·K), respectively, and the thermal conductivities of PEG/PCC and OD/PCC were 0.3178 W/(m·K) and 0.3413 W/(m·K), respectively. Compared to the pure PEG and OD, the thermal conductivities of PEG/PCC and OD/PCC were improved due to the addition of PCC-3-800 with a high graphite content. This was consistent with the results of studies [20,62] in the literature, in that the addition of biomass porous biomass carbon can promote an increase in thermal conductivity.

Figure 8a shows the temperature distribution images of OD, PEG, OD/PCC, and PEG/PCC during heating via a direct observation of infrared thermography to further evaluate their energy storage and heat transfer capacity during heating. The heating rate of OD was low from 0 min to 8 min, and the heating rate increased rapidly from 8 min to 10 min. The thermal energy storage of OD at about 30 °C limited the heating rate from 0 min to 8 min [10,15]. The low heating rate of OD/PCC indicated that the OD/PCC also underwent energy storage from 0 min to 4 min. The heating rate of PEG and PEG/PCC gradually decreased during heating, which was attributed to the fact that it became closer to the phase-change melting temperature and began to store thermal energy as the temperature increased, thereby reducing the heating rate. After heating for 10 min, the temperatures of OD and PEG increased to 54.2 °C and 41.7 °C, respectively, while the temperatures of OD/PCC and PEG/PCC increased to 61.4 °C and 60 °C, respectively. This meant that the heat transfer rates of PEG/PCC and OD/PCC were faster than those of PEG and OD, and their heat transfer capacities were improved, consistent with the experimental results of Figure 8c.

In order to investigate the solar–thermal conversion of OD, PEG, OD/PCC, and PEG/PCC, the samples were tested under a xenon lamp with simulated solar irradiation for 1000 s, and the results are shown in Figure 8b. The slow heating rate of PEG under xenon lamp irradiation indicated its weak solar–thermal conversion ability. At the same time, OD showed a phase transition plateau within the first 500 s of irradiation and rapid heating within 500–1000 s, which revealed that OD had a better solar–thermal conversion ability. Compared to OD and PEG, OD/PCC and PEG/PCC showed faster heating rates at the same time of irradiation, which displayed that the addition of PCC-3-800 could effectively improve the solar–thermal conversion capacity. During the heating process, OD/PCC and PEG/PCC showed phase transition plateaus at 8–200 s and 160–490 s due to their thermal energy storage. The solar thermal energy conversion efficiency (ŋ) was evaluated in Equation (9) [15,63].

(9) $$\eta =\frac{m\Delta H_{m,PCMs/PCC}}{PS(t_{t}-t_{f})}\times 100\%$$

where m is the mass of the prepared shape-stabilized OD/PCC and PEG/PCC, $\Delta H_{m,PCMs/PCC}$ is the melting enthalpy of the samples, P is the intensity of the simulated solar light irradiation (100 mW/cm^2^), S is the surface area of the samples, and $t_{t}$ and $t_{f}$ represent the starting and terminating times before and after the phase transition, respectively. According to the formula, the photothermal conversion efficiencies of PEG/PCC and OD/PCC were 79.9% and 84.8%, respectively, indicating the samples had an excellent photothermal conversion ability compared to the other composite phase-change materials in Figure 8d.

### 3.6. Compression Performance of Rigid Polyurethane Foam for Energy Storage

**Figure 9:**
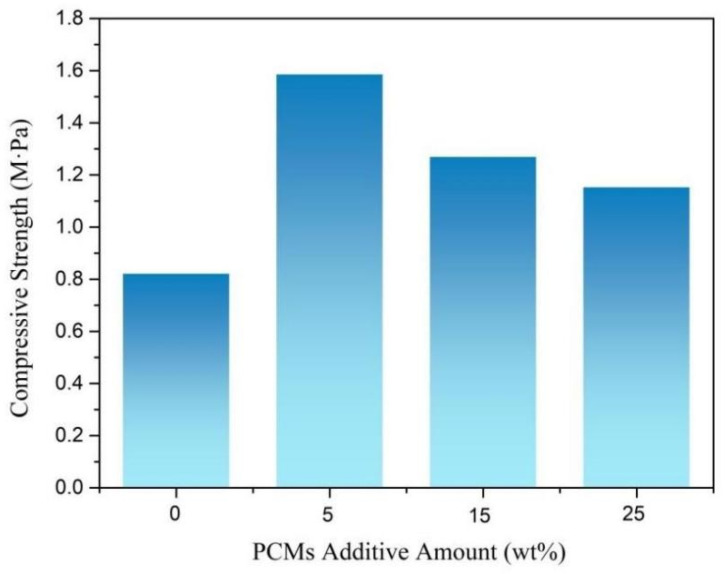
Compression properties of energy storage rigid polyurethane foams with different mass fractions of phase-change materials.

Figure 9 shows the compression performance of the rigid polyurethane foam in terms of energy storage with different mass fractions of phase-change materials. The compressive strength of the RPUF was 0.823 MPa, and the compressive properties of the polyurethane foam were improved when the phase-change material was added. During the process, part of PEG/PCC was broken, and the leaked PEG reacted with MDI to improve the compression properties of the material. However, with an increase in the addition of the phase-change materials, the compression properties of the rigid polyurethane foams declined [66,67]. Because the quantitative MDI was consumed after part of the leaked PEG reacted with MDI, PEG/PCC, OD/PCC, and all broken materials were used as fillers to affect the performance of the rigid polyurethane foam, and the mechanical properties decreased with an increase in the amount added.

### 3.7. Thermal Conductivity of Rigid Polyurethane Foam for Energy Storage

**Figure 10:**
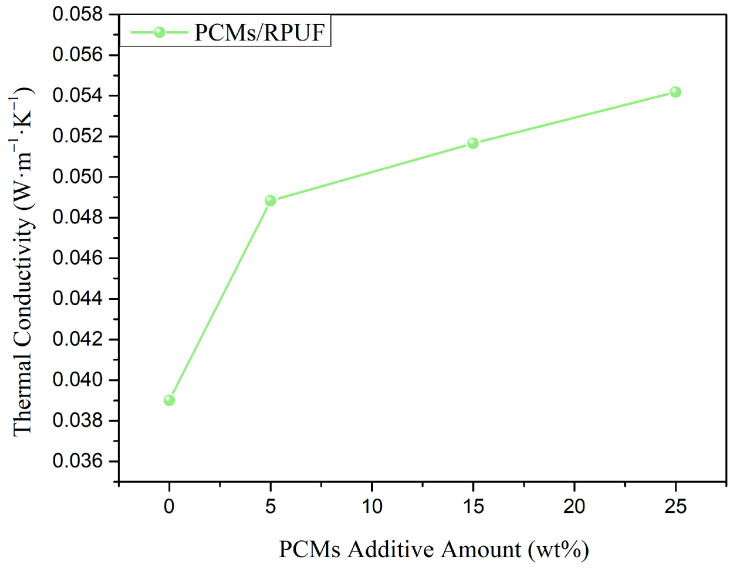
Thermal conductivity diagram of energy storage rigid polyurethane foams with different mass fractions of phase-change materials.

Thermal conductivity is an important parameter for evaluating the heat transfer in materials, and it also determines the rate at which thermal energy is stored and released. As shown in Figure 10, the thermal conductivity of the rigid polyurethane foam was 0.039 W/(m·K), and the thermal conductivities of PCMs/RPUF-5, PCMs/RPUF-15, and PCMs/RPUF-25 were 0.0488 W/(m·K), 0.0517 W/(m·K), and 0.0542 W/(m·K), respectively; compared to the RPUF, the thermal conductivity increased by 25.1%, 32.6%, and 39%. With an increase in the addition of the phase-change materials, the thermal conductivity of the rigid polyurethane foam for energy storage gradually increased, which was mainly due to the high thermal conductivities of the OD/PCC and PEG/PCC shape-stabilized phase-change materials, which could be added to the rigid polyurethane foam to improve the overall thermal conductivity of the polyurethane matrix.

### 3.8. Thermal Performance of Rigid Polyurethane Foam for Energy Storage

**Figure 11:**
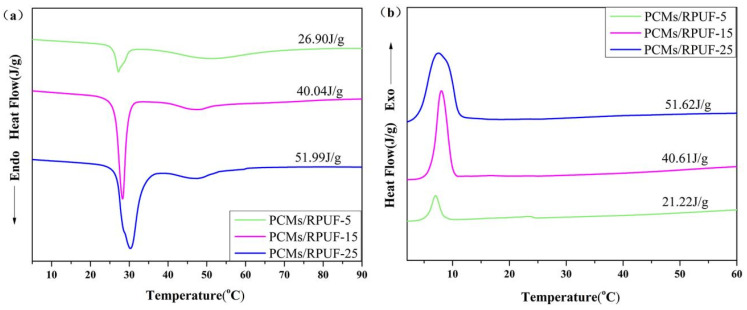
DSC diagram of energy storage rigid polyurethane foams with different mass fractions of phase-change materials: (**a**) melting curves and (**b**) crystallization curves.

In order to evaluate the performances of OD/PCC and PEG/PCC as thermal energy storage materials for building applications, the phase-change behavior of the energy-storing rigid polyurethane foam was studied by DSC, and the results are shown in Figure 11. PCMs/RPUF-5, PCMs/RPUF-15, and PCMs/RPUF-25 all had two endothermic peaks and two exothermic peaks, which were mainly provided by OD/PCC and PEG/PCC. The exothermic peak belonging to PEG/PCC was not apparent, mainly because part of the PEG/PCC leaked by stirring and crushing reacted with MDI, which further restricted the movement of PEG, and the content of PEG/PCC was relatively reduced, resulting in the enthalpy at the PEG/PCC phase transition temperature being lower. The melting enthalpies of PCMs/RPUF-5, PCMs/RPUF-15, and PCMs/RPUF-25 were 26.90 J/g, 40.04 J/g, and 51.99 J/g, respectively. Therefore, with an increase in the phase-change materials, the enthalpy of the energy-storing rigid polyurethane foam increased. The material had a great energy storage performance, stored energy at around 30 °C and 50 °C, and provided energy at around 8 °C and 23 °C, which could improve the energy utilization efficiency and is suitable for the field of green and energy-saving buildings.

**Figure 12:**
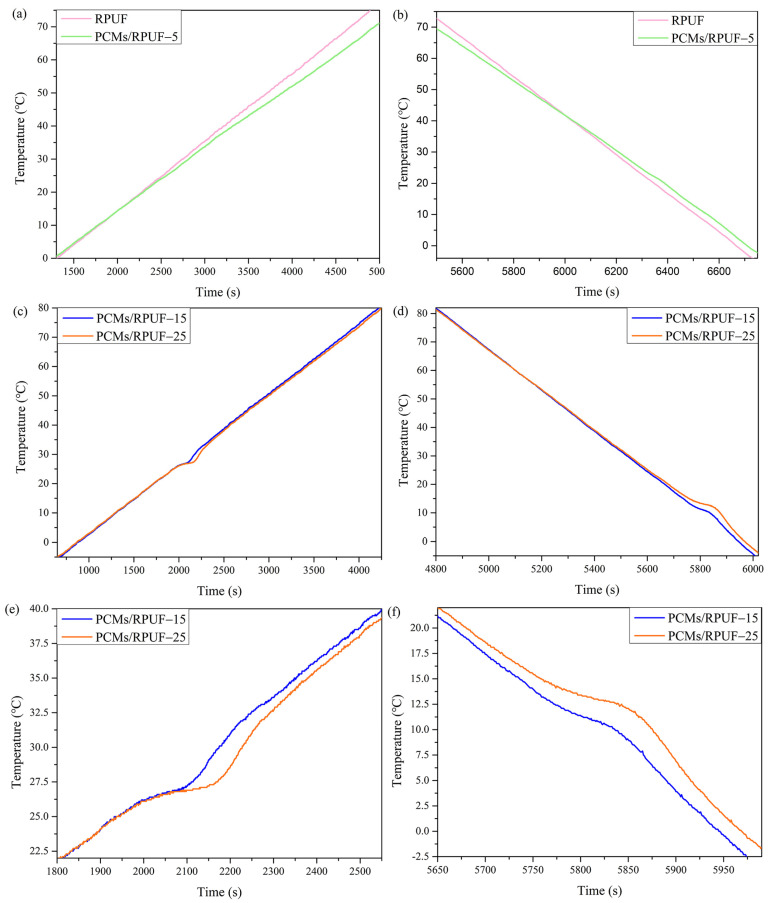
Temperature regulation diagram of energy-storing rigid polyurethane foams: (**a**,**b**) heating/cooling process of RPUF and PCMs/RPUF-5; (**c**,**d**) heating/cooling process of PCMs/RPUF-15 and PCMs/RPUF-25; and (**e**,**f**) enlarged diagrams of the warming/cooling phase transition process of PCMs/RPUF-15 and PCMs/RPUF-25.

In order to study the effect of the addition of the phase-change materials on the temperature-regulating and temperature-controlling capabilities of the rigid polyurethane foam, the sample was placed in an intelligent constant temperature tank for uniform heating and cooling, and the temperature of the sample was tested with time by using a thermocouple multi-channel temperature tester. In Figure 12a, the time-heating curve of the RPUF increased almost linearly, while the heating rate of PCMs/RPUF-5 decreased, which was mainly attributed to the energy storage capacities of OD/PCC and PEG/PCC. Therefore, after adding the phase-change materials, the time to reach the same temperature was extended during the heating process. When the temperature rose to 70 °C, the RPUF needed 4684 s, while the PCMs/RPUF-5 needed 4965 s, which was 281 s longer. It can be observed in Figure 12b that the time-cooling curve of the RPUF decreased almost linearly, while the cooling rate of PCMs/RPUF-5 was significantly lower than that of RPUF, which meant that OD/PCC and PEG/PCC released the previously stored energy and the cooling process also prolonged the time to reach the same temperature. The PCMs/RPUF-5 extended 43 s longer than the RPUF when the temperature was lowered to 0 °C. In Figure 12c,e, the PCMs/RPUF-15 and PCMs/RPUF-25 had prominent energy-absorbing constant temperature plateaus at 25 °C, and the constant temperatures were maintained for about 200 s and 250 s, respectively. In Figure 12d,f, the PCMs/RPUF-15 and PCMs/RPUF-25 had central constant temperature plateaus of energy release at 10 °C, and the constant temperature holding times were about 90 s and 131 s. In Figure 12c,d, it can also be seen that the heating rates of the PCMs/RPUF-15 and PCMs/RPUF-25 were further reduced from 40 °C during the heating process and the cooling rates of the PCMs/RPUF-15 and PCMs/RPUF-25 decreased from 27 °C during the cooling process, which was mainly attributed to the phase-change performance of PEG/PCC in the ridge polyurethane foam. Therefore, the temperature regulation and temperature control ability of the energy-storing rigid polyurethane foam was mainly endowed by the OD/PCC and PEG/PCC phase-change materials and gradually increased with an increase in the addition of the phase-change materials.

## 4. Conclusions

In this work, when the calcination temperature was 800 °C and the mass ratio of pine cones to KOH was 1:3, a three-dimensional interpenetrated network structure, including caterpillar-like and block-like structures with a high yield, was formed. The specific surface area was 1758.6 m^2^/g, the pore volume was 1.21 cm^3^/g, and the average pore diameter was 2.74 nm. The PEG/PCC and OD/PCC shape-stabilized phase-change composites had high enthalpies of 144.3 J/g and 162.3 J/g, respectively, and maintained an outstanding thermal reliability after 50 cycles of heating/cooling process. The thermal conductivities of PEG/PCC and OD/PCC were 0.3178 W/(m·K) and 0.3413 W/(m·K), respectively, and the photothermal conversion efficiencies reached 79.9% and 84.8%. When the total mass fraction of OD/PCC and PEG/PCC was 25 wt%, the compressive strength and enthalpy of the melting of the PCMs/RPUF-25 were 1.154 MPa and 51.99 J/g, respectively. The temperature-regulating and temperature-controlling capabilities of the energy-storing rigid polyurethane foam were gradually enhanced with an increase in the addition of the phase-change materials. There were obvious constant temperature platforms for energy absorption at 25 °C and for energy release at 10 °C. Therefore, PEG/PCC and OD/PCC shape-stabilized phase-change composites can be utilized in buildings and similar applications for thermal energy storage.

## Figures and Tables

**Table 1:** Denoted name of all PCC-X-Y samples.

Mass Ratio of Pine Cones to KOH	Calcination Temperature (°C)		
	700	800	900
1:1	-	PCC-1-800	-
1:2	PCC-2-700	PCC-2-800	PCC-2-900
1:3	PCC-3-700	PCC-3-800	PCC-3-900
1:4	-	PCC-4-800	-

**Table 2:** The mass fraction of OD/PCC and PEG/PCC corresponding to all sample names in rigid polyurethane foam.

Samples	RPUF	PCMs/RPUF-5	PCMs/RPUF-15	PCMs/RPUF-25
OD/PCC wt%	0	2.5	7.5	12.5
PEG/PCC wt%	0	2.5	7.5	12.5

**Table 3:** BET surface area and pore volume of PCC-X-Y and various carbon materials.

Samples	BET Surface Area (m^2^/g)	Total Pore Volume (cm^3^/g)	Mean Pore Size (nm)	Ref.
Rapeseed stem biochar	316.90	0.11	2.20	[41]
Activated garlic peel biomass carbon (AGP)	1309.00	0.54	2.20	[37]
Synthetic graphite-like carbon (Sib-4-ox-450 ^c^)	380.00	0.53	5.66	[42]
Synthetic graphite-like carbon (3%Ru/Sib-4-ox-450 ^c^)	341.00	0.50	5.88	[42]
Graphene-based metal and nitrogen-doped carbon (GNC-Co)	123.10	-	5.70	[43]
PCC-2-800	1405.40	0.55	1.58	This work
PCC-3-700	862.04	0.42	1.93	This work
PCC-3-800	1758.60	1.21	2.74	This work
PCC-3-900	679.73	0.38	2.26	This work

The “c” refers to the grains that are 56–94 µm in size.

**Table 4:** Thermal performance parameters of the samples.

Samples	T_m_ (°C)	ΔH_m_ (J/g)	T_c_ (°C)	ΔH_c_ (J/g)	R(%)
OD	27.5	194.5	24.6	193.3	-
PEG	56.1	157.8	34.9	151.9	-
OD/PCC	34.8	162.3	18.5	158.7	83.4
PEG/PCC	64.0	144.3	30.7	135.8	91.4

## Data Availability

Data is contained within the article.

## Supplementary Materials

The following supporting information can be downloaded at: https://www.mdpi.com/article/10.3390/polym15183747/s1, Figure S1: SEM images of PCC-800 (a), PCC-1-800 (b), PCC-4-800 (c). Figure S2: (a) Nitrogen adsorption/desorption isotherms and (b) DFT desorption pore size distribution of PCC-2-800. Figure S3: SEM images of composite PCMs: (a) PEG/PCC, and (b) OD/PCC. Figure S4: DSC curves of composite PCMs after thermal cycles: (a) PEG/PCC, and (b) OD/PCC. Table S1: BET surface area and pore volume of coniferous plants. Table S2: Thermogravimetric data of PCC. Table S3: Thermal transition capacity and the loading of recently reported composite PCMs. Table S4: Thermogravimetric data of PEG, OD, PEG/PCC and OD/PCC. References [68,69,70,71,72,73,74] are cited in the supplementary materials.
