# Supplementary material for: Biomass-Based Shape-Stabilized Composite Phase-Change Materials with High Solar–Thermal Conversion Efficiency for Thermal Energy Storage
Source: Polymers (Basel). 2023 Sep 13;15(18):3747. doi: 10.3390/polym15183747 (PMC10534447; doi:10.3390/polym15183747)
Supplement: Supplementary file 1 [file polymers-15-03747-s001.zip › polymers-2605424-supplementary.pdf]

Supporting Information

Biomass-Based Shape-Stabilized Composite Phase-Change Materials with High Solar–Thermal Conversion Efficiency for Thermal Energy Storage

Ning Gao [1], Jiaoli Du [1], Wenbo Yang [1], Youbing Li [1,*], Ning Chen [2,*]

[1] College of Materials Science and Engineering, Chongqing University of Technology, Chongqing 400054, China

[2] State Key Laboratory of Polymer Materials Engineering, Polymer Research Institute of Sichuan University, Chengdu 610065, China

* Correspondence: ningchen@scu.edu.cn, li-youbing@163.com

Figures

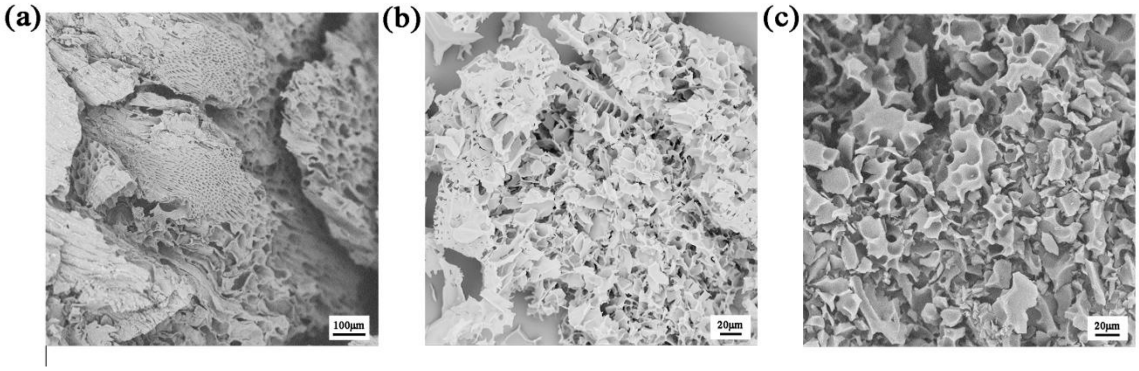

Figure S1. SEM images of PCC-800 (a), PCC-1-800 (b), PCC-4-800 (c).

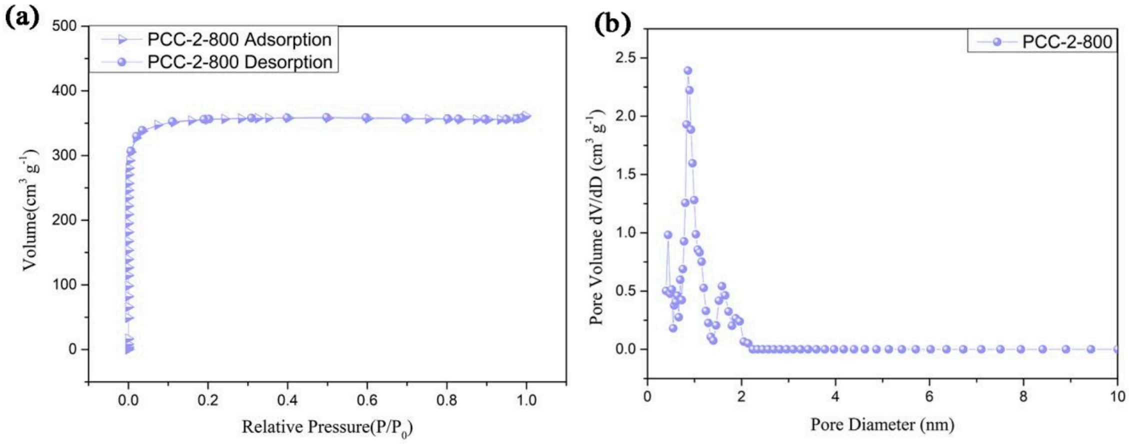

Figure S2. (a) Nitrogen adsorption/desorption isotherms and (b) DFT desorption pore size distribution of PCC-2-800.

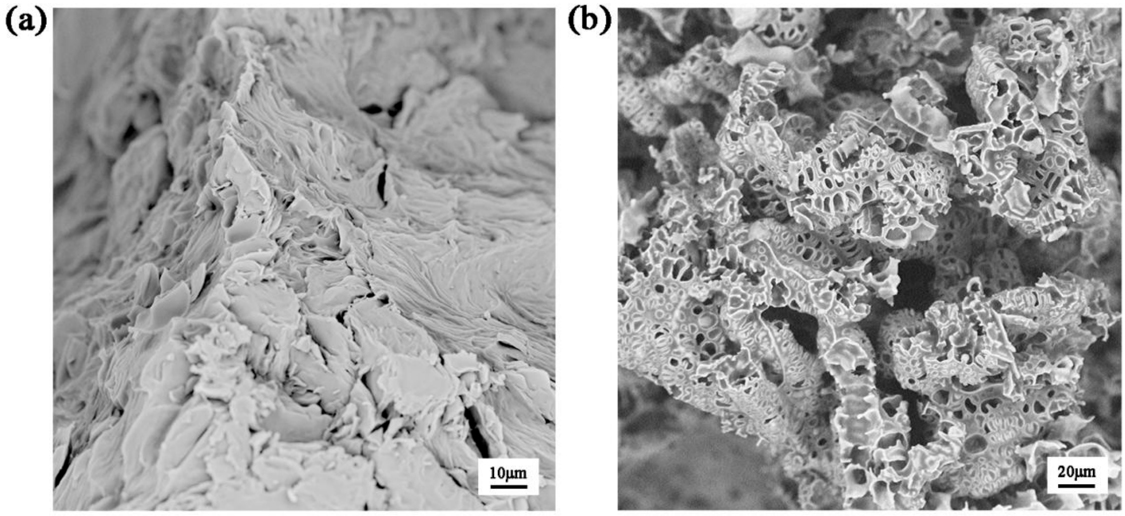

Figure S3. SEM images of composite PCMs: (a) PEG/PCC, and (b) OD/PCC.

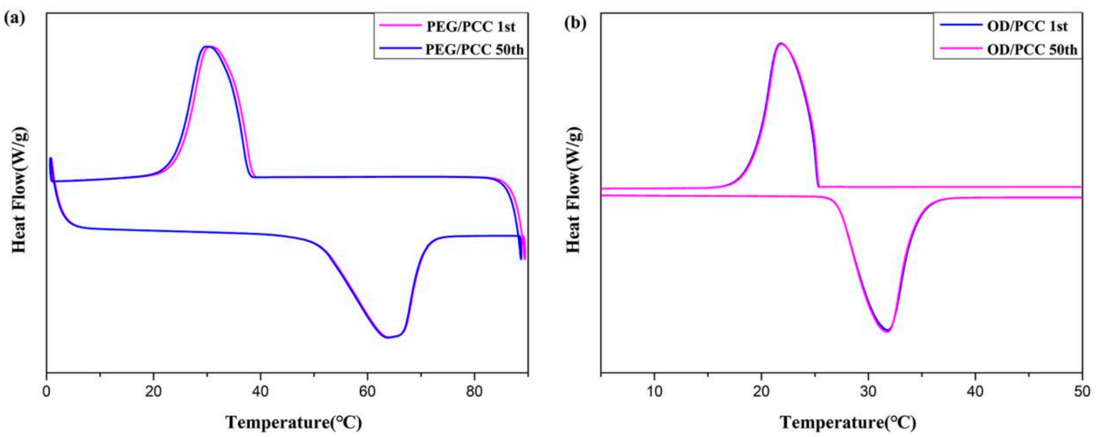

Figure S4. DSC curves of composite PCMs after thermal cycles: (a) PEG/PCC, and (b) OD/PCC.

Table

Table S1 BET surface area and pore volume of coniferous plants.

Samples	BET surface area (m^2/g)	Total pore volume (cm^3/g)	Pore size (nm)	Ref.
Cedarwood bark-derived carbon (CBC800)	441	0.226	1-3	[1]
Pine tannin-derived carbon (TW)	652	0.65	2-30	[2]
Pine tannin-derived carbon (TW-EA)	485	0.58	2-30	[2]
Pine tannin-derived carbon (TNa-A)	446	0.23	0.6-10	[2]
Pine tree seed carbon (PTAC13)	929.9	-	0.5-30	[3]
Carbonized pine	687.96	0.37	1.1-3	[4]
Dead pine needles-derived carbon (PN-1000)	783	0.49	2.55	[5]
PCC-2-800	1405.4	0.55	1.58	This work
PCC-3-700	862.04	0.42	1.93	This work
PCC-3-800	1758.60	1.21	2.74	This work
PCC-3-900	679.73	0.38	2.26	This work

Table S2. Thermogravimetric data of PCC.

Samples	$T_{5\%}$ (°C)	T_1 (°C)	T_2 (°C)	Mass at 800°C (%)
PCC-3-700	285	152	412	85.2
PCC-3-800	559	185	460	90.9
PCC-3-900	431	164	465	92.4

Table S3. Thermal transition capacity and the loading of recently reported composite PCMs.

Supporting materials	PCMs	Latent heat (J/g)	Loading (wt%)	η (%)	Ref.
Pinecone biochar	Palmitic acid	84.7	38.6	-	[6]
Pinus resinosa biochar	Dodecanoic acid	50.87	32.53	-	[7]
Softwood biochar	Eicosane	53.4	26.4	-	[8]
Wheat straw biochar	Eicosane	75	37.1	-	[8]
Potato derived carbon	PEG4000	91.8	50.0	-	[9]
PCC-3-800	PEG4000	144.26	91.41	79.9	This work
PCC-3-800	Octadecane	162.25	83.44	84.8	This work

Table S4. Thermogravimetric data of PEG, OD, PEG/PCC and OD/PCC.

Samples	$T_{5\%}$ (°C)	Tmax (°C)	Mass at 800°C (%)
PEG	372	427	0
OD	141	229	0
PEG/PCC	352	435	5.1
OD/PCC	142	476	22.7

References

[1] Lu, M.; Huang, Y.; Chen, C. Cedarwood bark-derived hard carbon as an anode for high-performance sodium-ion batteries. Energy Fuels 2020, 34, 11489-11497.

[2] Sanchez-Sanchez, A.; Izquierdo, M.T.; Mathieu, S.; González-Álvarez, J.; Celzard, A.; Fierro, V. Outstanding electrochemical performance of highly N-and O-doped carbons derived from pine tannin. Green Chem. 2017, 19, 2653-2665.

[3] Murali, G.; Kesavan, T.; Ponnusamy, S.; Harish, S.; Navaneethan, M. Improved supercapacitor performance based on sustainable synthesis using chemically activated porous carbon. J. Alloys Compd. 2022, 906, 164287.

[4] He, R.; Neupane, M.; Zia, A.; Huang, X.; Bowers, C.; Wang, M.; Lu, J.; Yang, Y.; Dong, P. Binder-free wood converted carbon for enhanced water desalination performance. Adv. Funct. Mater. 2022, 32, 2208040.

[5] Leng, C.; Sun, K.; Li, J.; Jiang, J. From dead pine needles to O, N codoped activated carbons by a one-step carbonization for high rate performance supercapacitors. ACS Sustain. Chem. Eng. 2017, 5, 10474-10482.

[6] Wan, Y.; Chen, Y.; Cui, Z.; Ding, H.; Gao, S.; Han, Z.; Gao, J. A promising form-stable phase change material prepared using cost effective pinecone biochar as the matrix of palmitic acid for thermal energy storage. Sci. Rep. 2019, 9, 11535.

[7] Mandal, S.; Ishak, S.; Lee, D.E.; Park, T. Optimization of eco-friendly Pinus resinosa biochar-dodecanoic acid phase change composite for the cleaner environment. J. Energy Storage 2022, 55, 105414.

[8] Atinafu, D.G.; Yun, B.Y.; Kim, Y.U.; Wi, S.; Kim, S. Introduction of eicosane into biochar derived from softwood and wheat straw: Influence of porous structure and surface chemistry. Chem. Eng. J. 2021, 415, 128887.

[9] Tan, B.; Huang, Z.; Yin, Z.; Min, X.; Liu, Y.G.; Wu, X.; Fang, M. Preparation and thermal properties of shape-stabilized composite phase change materials based on polyethylene glycol and porous carbon prepared from potato. RSC Adv. 2016, 6, 15821-15830.
